# The HMGB1–RAGE axis in nucleus accumbens facilitates cocaine‐induced conditioned place preference via modulating microglial activation

**DOI:** 10.1002/brb3.3457

**Published:** 2024-03-07

**Authors:** Jian Ye, Shuang‐Qi Gao, Zi‐Cun Liu, Xi Chen, Jin‐Gang He, Zhuang‐Li Hu

**Affiliations:** ^1^ Department of Pharmacology, School of Basic Medicine, Tongji Medical College Huazhong University of Science and Technology Wuhan China; ^2^ The Key Laboratory for Drug Target Researches and Pharmacodynamic Evaluation of Hubei Province Wuhan China; ^3^ The Research Center for Depression, Tongji Medical College Huazhong University of Science and Technology Wuhan China; ^4^ Departments of Neurosurgery Third Affiliated Hospital of Sun Yat‐Sen University Guangzhou Guangdong Province China

**Keywords:** cocaine addiction, conditioned place preference, high mobility group box‐1, microglia, receptor for advanced glycation end products

## Abstract

**Introduction:**

Repeated exposure to cocaine induces microglial activation. Cocaine exposure also induces a release of high mobility group box‐1 (HMGB1) from neurons into the extracellular space in the nucleus accumbens (NAc). HMGB1 is an important late inflammatory mediator of microglial activation. However, whether the secretion of HMGB1 acts on microglia or contributes to cocaine addiction is largely unknown.

**Methods:**

Rats were trained by intraperitoneal cocaine administration and cocaine‐induced conditioned place preference (CPP). Expression of HMGB1 was regulated by viral vectors. Activation of microglia was inhibited by minocycline. Interaction of HMGB1 and the receptor for advanced glycation end products (RAGE) was disrupted by peptide.

**Results:**

Cocaine injection facilitated HMGB1 signaling, together with the delayed activation of microglia concurrently in the NAc. Furthermore, the inhibition of HMGB1 or microglia activation attenuated cocaine‐induced CPP. Box A, a specific antagonist to interrupt the interaction of HMGB1 and RAGE, abolished the expression of cocaine reward memory. Meanwhile, the inhibition of HMGB1–RAGE interaction suppressed cocaine‐induced microglial activation, as well as the consolidation of cocaine‐induced memory.

**Conclusion:**

All above results suggest that the neural HMGB1 induces activation of microglia through RAGE, which contributes to the consolidation of cocaine reward memory. These findings offer HMGB1–RAGE axis as a new target for the treatment of drug addiction.

## Graphical abstract

The formation of cocaine memory was mediated by cocaine binding directly to TLR4, and the consolidation of cocaine memory was induced by HMGB1–RAGE axis. RAGE was engaged secondary to (TLR4) after cocaine exposure and involved in the activation of microglia. There was a neural HMGB1‐dependent role for cocaine‐induced microglial activation in the NAc through RAGE, which contributed to the consolidation of cocaine reward memory.

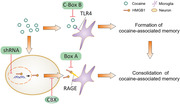

## 1 INTRODUCTION

Drug abuse constitutes a serious global social and health concern. With respect to drug abuse, cocaine is the most reinforcing drug that causes addiction. Cocaine strongly enhances brain reward system, and compulsory withdrawal causes forced drug‐seeking behavior and even drug relapse. More recent studies have confirmed that cocaine is a potent activator of microglia (Burkovetskaya et al., 2020; Kumar et al., 2020; Linker et al., 2020) and induces microglia to activate innate immune response in the brain (da Silva et al., 2023; Kashima & Grueter, 2017; Northcutt et al., 2015; Wang et al., 2016). Minocycline, a widely used inhibitor of microglial activation, inhibits cocaine‐induced locomotor sensitization and self‐administration (Avalos et al., 2022; Linker et al., 2020). In addition, many studies have found that toll‐like receptor 4 (TLR4) is involved in the development of addictive behaviors. Cocaine even directly binds to microglial TLR4, and blocking TLR4 with (+)‐naloxone or (+)‐naltrexone can prevent the expression of cocaine‐induced adaptation behaviors in animal models (Northcutt et al., 2015). However, there is no complete loss of cocaine reward learning in TLR4 KO mice (Kashima & Grueter, 2017), and the TLR4 antagonists cannot specifically block neurochemical or behavioral abuse‐related effects of cocaine (Tanda et al., 2016). So far, the related therapeutic approaches are retarded as the underlying mechanisms and therapeutic targets remain unclear.

High mobility group box‐1 (HMGB1), a highly conserved nonhistone nuclear protein, contributes to various aspects of nuclear homeostasis, such as stabilization of nucleosomes, DNA repair and recombination, and gene transcription (Andersson & Tracey, 2011; Harris et al., 2012; Malarkey & Churchill, 2012; Xue et al., 2021). Serving as a damage‐associated molecular pattern (DAMP) (Chen, Kang, et al., 2022; Harris et al., 2012), it can be transferred to cytosol and released into the extracellular space. HMGB1 consists of three structural domains, termed “Box A (HMGB11‐79),” “Box B (HMGB189‐163),” and a negatively charged carboxyl terminus (HMGB1186‐215) (Andersson & Tracey, 2011; Harris et al., 2012; Mao et al., 2022). Moreover, it has been previously shown that the Box B recapitulates the pro‐inflammatory activity, whereas the Box A acts as an antagonist of HMGB1 (Andersson & Tracey, 2011; Muhammad et al., 2008). Indeed, the biological and clinical importance of HMGB1 has been underscored in multiple pathological conditions, including sepsis, ischemia–reperfusion injury, arthritis, Parkinson's disease, epilepsy, and cancer (Andersson & Tracey, 2011; Harris et al., 2012; Mao et al., 2022; Xue et al., 2021). Recent evidence suggests that HMGB1 plays a role in drug addiction (Frank et al., 2016; Masai et al., 2021; Scobie et al., 2014; Vannier et al., 2021). Similarly, we have reported that neuronal HMGB1 in nucleus accumbens (NAc) regulates the formation of cocaine reward memory (Gao et al., 2020). After secretion, extracellular HMGB1 will trigger a series of reactions, especially immune and inflammatory responses through binding to different receptors (Andersson & Tracey, 2011; Mo et al., 2023; Xue et al., 2021). Many receptors of HMGB1, such as TLR2, TLR4, TLR9, the receptor for advanced glycation end products (RAGE), and so on, are primarily distributed on microglia, which are tissue‐resident immune cells in the brain (Franklin et al., 2018; Kashima & Grueter, 2017; Lehnardt, 2010; Su et al., 2016). Among them, TLR4 and RAGE are the main two receptors that share common ligand HMGB1 (Andersson & Tracey, 2011; Harris et al., 2012). TLR4 even mediates the activation of microglia via interacting with cocaine directly. However, the different mechanisms between TLR4 and RAGE receptors contributing to the onset and relapse of drug remain to be elucidated, particularly the role of RAGE in cocaine‐related addictive behavior is still largely unknown.

In the present study, we demonstrate that HMGB1 mediates the late effect of cocaine‐induced microglial activation by HMGB1–RAGE axis, which facilitates consolidation of cocaine‐induced conditioned place preference (CPP). These findings provide further evidence that cocaine produces addiction‐like behaviors by acting on neuroimmune signaling pathway and offers a new therapeutic target for the drug addiction treatment.

## 2 MATERIALS AND METHODS

### 2.1 Animals

Male Sprague Dawley rats weighing 250–350 g were used in the present study. The rats were housed on a controlled 12 h light cycle at a constant temperature (22 ± 2°C) and humidity of 50% ± 10% with food and water provided ad libitum at all times unless specified. Unless otherwise noted, all rats were housed with littermates in groups of two to four and were used for only one behavioral test apiece.

### 2.2 Western blotting

Tissue preparations and western blots were conducted as previously described (Gao et al., 2020). Briefly, NAc and medial prefrontal cortex (mPFC) samples were lysed in ice‐cold lysis buffer (in mM) (50 Tris–HCl, 1 EDTA, 100 NaCl, 20 NaF, 3 Na_3_VO_4_, 1 phenylmethanesulfonyl fluoride, 1% Nonidet P‐40 (v/v), with 10% protease inhibitor (v/v)) (Ameresco). Protein extracts were denaturated with SDS–PAGE sample buffer that with β‐mercaptoethanol and 95°C/5 min. Equal amounts of protein (10–30 μg) from above lysates were loaded in each lane and separated on 10% or 12% SDS–PAGE gels and then transferred to nitrocellulose membranes (transfer buffer: 25 mM Tris, 190 mM glycine, 20% methanol, 0.5% SDS). The membranes were washed in Tris‐buffered saline (TBS 20 mM, Tris–HCl pH 7.6, 140 mM NaCl) and blocked with 5% BSA in TBS containing 0.5% Tween 20 (TBS‐T) as described above. After incubating overnight at 4°C with the primary antibodies (Table S1), membranes were washed with TBS‐T solution and then incubated for 60 min with horseradish peroxidase‐conjugated anti‐rabbit IgG or anti‐mouse IgG (1:10,000 dilutions; Pierce Chemical). After that, the membranes were washed with TBS‐T, rinsed with double‐deionized water, and immersed in enhanced chemiluminescence detecting substrate (Super Signal West Pico; Pierce Chemical). Images were captured with Micro Chemi (DNR Bio‐Imaging Systems). The pictures were scanned, and the optical density of the bands was determined using NIH ImageJ software.

### 2.3 Conditioned place preference (CPP)

The CPP was carried out as previously described (Gao et al., 2020). Place conditioning was conducted in a Plexiglas apparatus (Context A: 25 × 25 × 30 cm^3^ or Context B: 24 × 20 × 30 cm^3^) with a sound‐attenuating lid (AniLab). There was a removable center divider that separated the apparatus into two compartments with equal size. Each side of the apparatus differed in wall pattern and floor texture. All experiments were conducted during the light cycle.

On conditioning sessions (45 min; at 8:00–12:00), the rats were confined to one compartment. On testing sessions (15 min; at 12:00–14:00), the rats were allowed to freely access to both compartments through a divider with a door. Both dividers were opaque and colored on each side to match the compartment‐facing wall pattern, marble, or wood, respectively. To assess baseline preferences, pretesting sessions were conducted (pretest CPP) in which naive rats were placed individually in the CPP apparatus and allowed to freely explore for 15 min. Any rats that spent <40% or >60% of the entire time in either environment were removed from the study (Fan et al., 2015). For conditioning sessions, rats were given saline (SAL) injection (1 mL/kg) and confined to one compartment (days 1, 3, 5, and 7) or received cocaine injection (15 mg/kg, intraperitoneal [i.p.]) and confined to the other compartment (days 2, 4, 6, and 8). The CPP score was defined as the time (in seconds) spent in the cocaine‐paired chamber minus the time spent in the SAL‐paired chamber during CPP testing (posttest CPP) (Fan et al., 2015).

For detection the effect of minocycline (Mino) hydrochloride, carbenoxolone (CBX), and glycyrrhizin (GL), pre‐exposure was conducted to assess baseline preferences as described above. For conditioning, rats received i.p. of (1) 30 mg/kg Mino, (2) 10, 20, and 40 mg/kg CBX, and (3) 50 mg/kg GL or vehicle (SAL) 45 min before the administration of 15 mg/kg cocaine or SAL. In this case, rats experienced four cycles of conditioning sessions. Place preference was assessed on 9th day as described above, and all rats were tested in a drug‐free state. In some CPP experiments, after daily conditioning sessions, rats received bilateral intra‐NAc infusion (2.5 μL per side, 0.5 μL/min) of (1) Box A (5 μg), (2) C106‐Box B or S106‐Box B (10 μg) or vehicle (phosphate‐buffered saline, PBS). In this case, rats experienced two cycles of conditioning sessions. Place preference was assessed on 3rd day as described above, and all rats were tested in a drug‐free state (Boury‐Jamot et al., 2016). In some CPP experiments, before 15 min or 2 days daily conditioning sessions, rats received bilateral intra‐NAc infusion (2.5 μL per side, 0.5 μL/min) of C106‐Box B or S106‐Box B (10 μg) or PBS. In this case, rats experienced two or one cycles conditioning sessions. Place preference was assessed on 3rd or 4th day as described above, and all rats were tested in a drug‐free state (Boury‐Jamot et al., 2016).

### 2.4 Statistical analysis

Data were analyzed with the statistical program SPSS 18.0 software (SPSS). Comparison between two independent groups was evaluated by an unpaired Student's *t*‐test. Differences in different treatment groups were evaluated by one‐ or two‐way ANOVAs followed by Bonferroni's post hoc test. Data were presented as mean ± SEM (significant at *p* < .05).

Full detail methods of systemic administration or stereotaxic injection of drugs and immunohistochemistry were available in Supplementary Information section.

## 3 RESULTS

### 3.1 Repeated cocaine exposure induces the activation of microglia and increases the expression of TLR4 in the NAc

**FIGURE 1:**
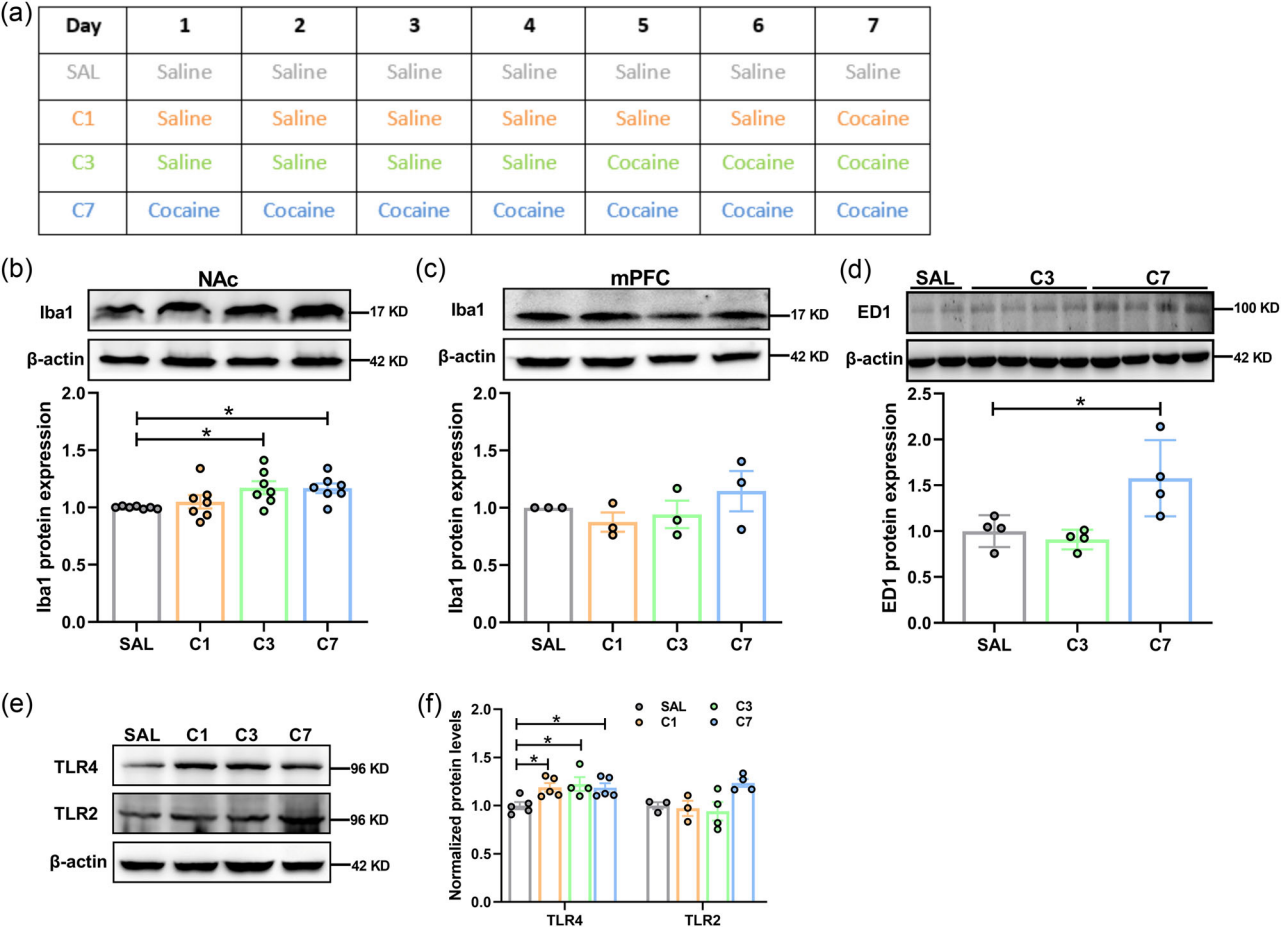
Repeated cocaine exposure induces the activation of microglia and increases the expression of toll‐like receptor 4 (TLR4) in the nucleus accumbens (NAc). (a) Schematic representations of repeated cocaine exposure, saline (SAL), or cocaine (15 mg/kg) were intraperitoneally administered to rats. (b and c) Western blots showing the expression of Iba1 in NAc (b) (*n* = 7 rats per group) and in medial prefrontal cortex (mPFC) (C) (*n* = 3 rats per group) after SAL or cocaine administration. (d) Western blots showing the expression of ED1 in NAc after SAL or cocaine administration (*n* = 4 rats per group). (e and f) Western blotting showing the protein levels of TLRs in NAc of rats with SAL or cocaine injection (TLR4: *n* = 5 saline (SAL), 5 C1, 4 C3, and 5 C7 rats; TLR2: *n* = 3 SAL, 3 C1, 4 C3, and 4 C7 rats). All data are presented as the mean ± SEM, with each point representing data from an individual. One‐way ANOVA followed by Bonferroni's post hoc test for (B–D, F), *p < .05. See Table S4 for detailed statistical information.

Microglia are the resident immunocompetent cells and the major modulators of inflammation in the brain, their activation plays a significant role in cocaine action (da Silva et al., 2023; Lewitus et al., 2016; Northcutt et al., 2015; Thangaraj et al., 2020). We thus examined microglia alterations in the mPFC and NAc after cocaine exposure (Figure 1a–c). The expression of ionized calcium‐binding adapter molecule 1 (Iba1), a microglia‐specific marker (Dwir et al., 2020), was significantly increased in the NAc (Figure 1b), but not in the mPFC (Figure 1c) at 24 h after three or seven daily injections of cocaine (C3 or C7) relative to SAL‐treated rats. Furthermore, ED1 (CD68), a marker of reactive microglia and macrophages (Dwir et al., 2020), was also increased in the NAc with a delay time at C7 (Figure 1d). These results suggest that the activation of microglia in the NAc induced by cocaine has a specific time window.

HMGB1 is an important late inflammatory mediator of microglial activation that is induced by cortical spreading depression (Karatas et al., 2013; Takizawa et al., 2017), stress (Chen, Jiang, et al., 2022; Franklin et al., 2018), or inflammation (Yang et al., 2022). HMGB1 is classified as three forms: all‐thiol‐HMGB1, disulfide‐HMGB1, and oxidized‐HMGB1, which have different functions (Venereau et al., 2012; Xue et al., 2021). We further investigated the alteration of HMGB1 after cocaine exposure. Principally, polyclonal antibodies against HMGB1 recognized all forms of HMGB1. As the samples were heated at 95°C without β‐mercaptoethanol, and HMGB1 was shifted to the oxidized‐HMGB1 (Figure S1A,B), the result illustrated that the oxidative type of HMGB1 was tended to increase after cocaine treatment.

Extracellular HMGB1 exists mainly with the oxidative type of HMGB1, which can bind to different receptors to promote a variety of cellular responses (Raucci et al., 2019). As the major binding partners of HMGB1, TLRs are predominantly expressed on microglia (Harris et al., 2012). We then determined whether TLR signal was involved in cocaine action. We first examined the protein expression of two common HMGB1 receptors TLR2 and TLR4 (Harris et al., 2012) in the NAc after cocaine exposure. It was found that TLR4, but not TLR2, was maintained at a high level during cocaine exposure (Figure 1e,f). These results suggest that TLR4 is upregulated in the NAc after cocaine exposure, indicating that TLR4 signaling pathway may be involved in the formation and consolidation of cocaine‐associated memory.

### 3.2 Extracellular HMGB1 and microglial activation are required for cocaine‐induced CPP

**FIGURE 2:**
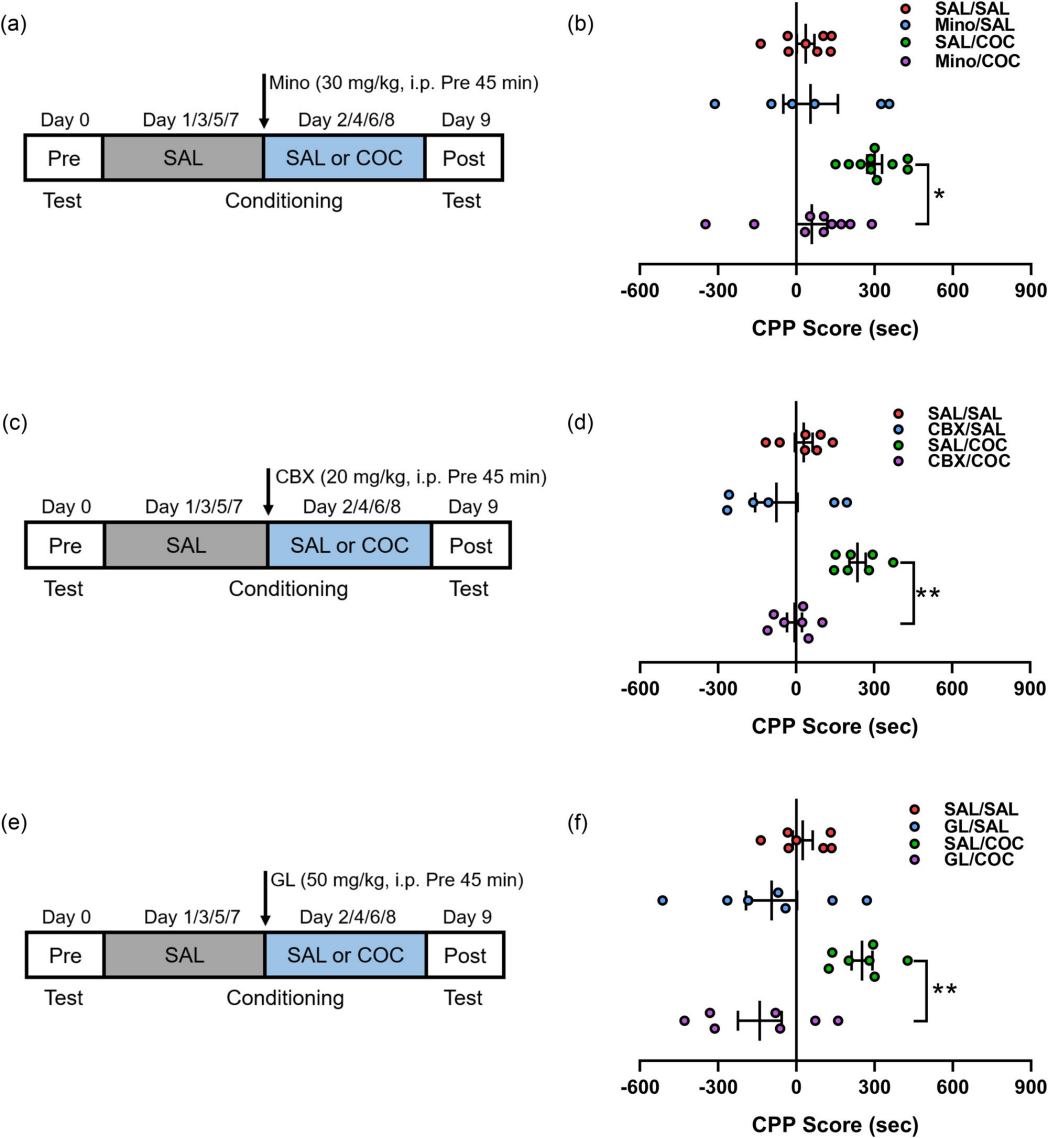
Blockade of high mobility group box‐1 (HMGB1) expression and secretion or inhibition of microglial activation attenuates cocaine‐induced conditioned place preference (CPP). (a and b) Intraperitoneal injection of saline (SAL) or minocycline (Mino) into rats followed by training sessions and the altered CPP score with 30 mg/kg Mino pretreatment (*n* = 8 SAL/SAL, 6 Mino/SAL, 10 SAL/cocaine [COC], and 10 Mino/COC rats). (c and d) Intraperitoneal injection of saline (SAL) or carbenoxolone (CBX) into rats and the altered CPP score with 20 mg/kg CBX pretreatment (*n* = 7 SAL/SAL, 6 CBX/SAL, 7 SAL/COC, and 7 CBX/COC rats). (e and f) Intraperitoneal injection of saline (SAL) or glycyrrhizin (GL) into rats and the altered CPP score with 50 mg/kg GL pretreatment (*n* = 7 rats per group). All data are presented as the mean ± SEM, with each point representing data from an individual. Two‐way ANOVA followed by Bonferroni's post hoc test for (B, D, F). *p < .05, **p < .01. See Table S4 for detailed statistical information.

In order to determine the impact of microglial activation on cocaine‐induced CPP of adult rats, Mino (30 mg/kg), a classical inhibitor of microglial activation that can penetrate the blood–brain barrier (Cutando et al., 2013; Northcutt et al., 2015) was administered via i.p. injection (Cutando et al., 2013; Figueiredo, 2019; Hui et al., 2019) (Figure 2a). The behavioral results showed that cocaine administration produced obvious CPP, which was suppressed by the pretreatment of Mino (Figure 2b), indicating that the activation of microglia is participated in cocaine‐induced CPP.

We have reported that repeated cocaine exposure induces HMGB1 expression and secretion from neurons in the NAc (Gao et al., 2020). To investigate whether HMGB1 secretion contributed to cocaine‐induced CPP, CBX, a pannexin‐1 channel blocker (Karatas et al., 2013) was used to inhibit HMGB1 release (Hisaoka‐Nakashima et al., 2020). The suppressive effect of CBX at different concentrations indicated that obvious CPP induced by cocaine administration could be restrained by the pretreatment of CBX in a dose‐dependent manner at 20 and 40 mg/kg, but not at 10 mg/kg (Figure 2c,d; Figure S2A,B). Furthermore, GL (50 mg/kg), an inhibitor that physically binds to HMGB1, was used to prevent HMGB1 extracellular activity (Shinde‐Jadhav et al., 2021) (Figure 2e), and the behavioral results showed that cocaine administration produced obvious CPP, which was suppressed by the pretreatment of GL (Figure 2f). Taken together, these results demonstrate that both extracellular HMGB1 and microglial activation are required for cocaine‐induced CPP.

### 3.3 Microglial activation is dependent on non‐microglial HMGB1 secretion during cocaine‐induced CPP

**FIGURE 3:**
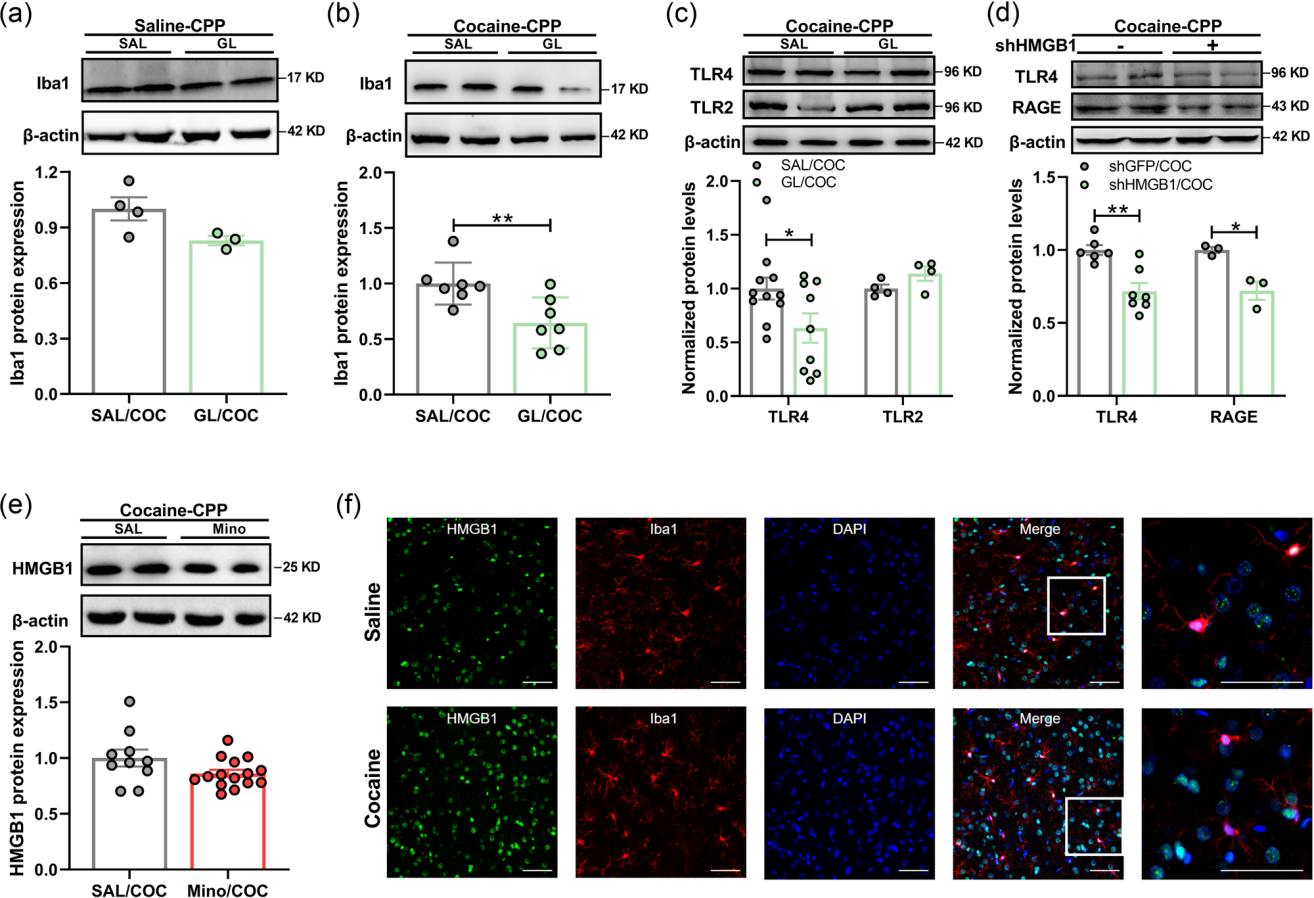
Microglial activation is dependent on non‐microglial high mobility group box‐1 (HMGB1) secretion in the nucleus accumbens (NAc) during cocaine‐induced conditioned place preference (CPP). (a and b) Western blotting showing that the level of Iba1 was decreased by glycyrrhizin (GL) pretreatment during cocaine‐induced CPP (B) (*n* = 7 rats per group) but not saline (SAL)‐CPP (a) (*n* = 4 saline (SAL)/cocaine [COC] and 3 GL/COC rats). (c) Western blots showing altered expression of toll‐like receptor 4 (TLR4) and TLR2 in the NAc of rats during cocaine‐induced CPP with SAL or GL pretreatment (TLR4: *n* = 11 SAL/cocaine [COC], *n* = 9 GL/COC rats; TLR2: *n* = 4 rats per group). (d) Western blots showing altered expression of TLR4 and receptor for advanced glycation end products (RAGE) in the NAc of rats after posttest CPP with shGFP or shHMGB1 treatment (TLR4: *n* = 6 shGFP/COC, *n* = 7 shHMGB1/COC rats; TLR2: *n* = 3 rats per group). (e) Western blotting showing that the level of HMGB1 was not decreased by Mino pretreatment during cocaine‐induced CPP (*n* = 10 SAL/COC, *n* = 15 Mino/COC rats). (f) Representative confocal micrographs of double‐immunofluorescence staining for HMGB1 (green) and Iba1 (red) in the NAc of SAL and cocaine exposure rats after context paired conditioning (*n* = 3 rats × 3 slices per group, scale bars = 50 μm). All data are presented as the mean ± SEM, with each point representing data from an individual. Student's *t* test for (A–E). *p < .05, **p < .01. See Table S4 for detailed statistical information.

Next, NAc core region was taken out (Figure S3A) to examine whether there was a correlation between HMGB1 and activation of microglia during cocaine‐induced CPP. Considering that GL could inhibit extracellular activity of HMGB1 via binding to HMGB1 (Paudel et al., 2020; Shinde‐Jadhav et al., 2021), especially reducing HMGB1–RAGE interaction (Okuma et al., 2014), GL was used to determine the effect of HMGB1 on microglial activation during cocaine‐induced CPP. Although there was no effect of GL on microglial activation in SAL‐treated mice (Figure 3a), GL suppressed cocaine‐induced increased level of Iba1 (Figure 3b). Furthermore, pretreatment with GL (Figure 3c) or knockdown HMGB1 by LV‐shHMGB1 (Figure 3d) attenuated the TLR4/RAGE signaling pathway during cocaine‐induced CPP, indicating that HMGB1–TLR4/RAGE‐microglia was involved in cocaine‐related learning and memory. However, different from parallel effects of GL, Mino did not reverse the cocaine‐induced upregulation of HMGB1 in the NAc (Figure 3e). Next, we detected the distribution of HMGB1 at 24 h after final conditioning, with DAPI for visualizing nuclei, as well as the antibody against Iba1, a microglia marker. There was no change of HMGB1 distribution in the nuclei of microglia in context‐paired rats relative to controls (Figure 3f); thus, the extracellular HMGB1 was not originated from microglia. These results demonstrated that cocaine could activate microglia by acting on HMGB1 in the NAc, contributing to addictive behaviors.

### 3.4 HMGB1–TLR4 signaling pathway does not contribute to cocaine‐associated memory formation

**FIGURE 4:**
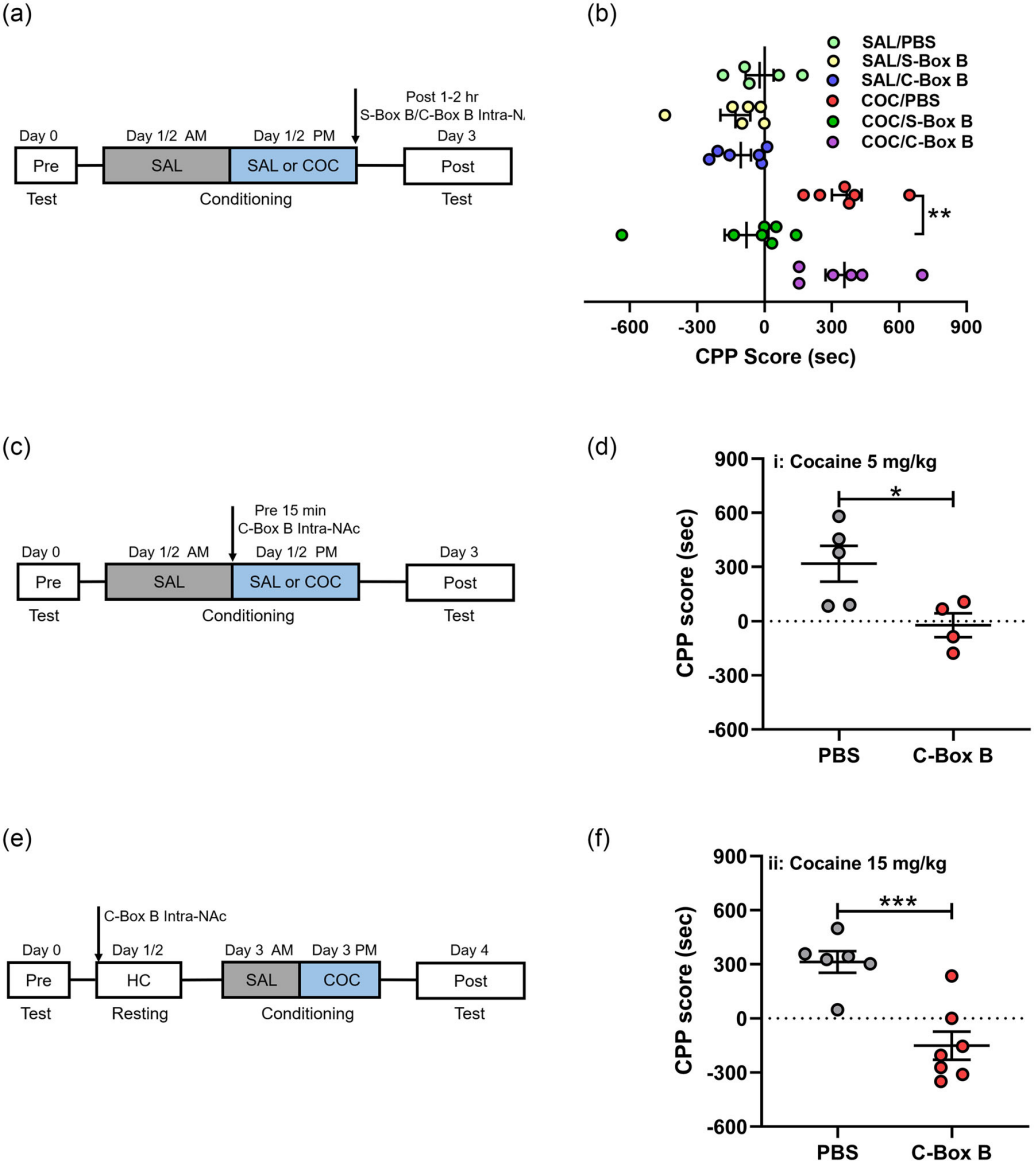
The cocaine‐toll‐like receptor 4 (TLR4) signaling in the nucleus accumbens (NAc) contributes to the formation of cocaine memory. (a) Schematic representation of injection of C‐Box B or S‐Box B into the NAc of rats followed by training sessions. (b) The altered conditioned place preference (CPP) score with C‐Box B or S‐Box B treatment (*n* = 5‐7 rats per group). (C and D) Timeline of the experiment (c) and the CPP score (d) after pretreatment with phosphate‐buffered saline (PBS) or C‐Box B into the NAc of rats at 5 mg/kg cocaine (*n* = 4–5 rats per group). (E and F) Timeline of the experiment (e) and the CPP score (f) after pretreatment with PBS or C‐Box B into the NAc of rats at 15 mg/kg cocaine (*n* = 6–7 rats per group). All data are presented as the mean ± SEM, with each point representing data from an individual. Two‐way ANOVA followed by Bonferroni's post hoc test for (B) and Student's *t* test for (D, F). *p < .05, ***p < .001. See Table S4 for detailed statistical information.

Previous studies have shown that both cocaine (Northcutt et al., 2015) and HMGB1 (Xu et al., 2020) interact with TLR4 on the surface of microglial cells, and cocaine‐induced TLR4 signaling in microglia is necessary for the cocaine‐induced CPP. However, the role of HMGB1–TLR4 signaling in cocaine‐induced CPP is still unknown. Then, we investigated the role of HMGB1–TLR4 signaling in vivo. As cysteine (C) 106‐Box B of HMGB1 mediates the binding to TLR4 (Yang et al., 2010), we designed the peptide of C106‐Box B (C‐Box B) to mimic the role of HMGB1/TLR4 signaling, and S106‐Box B (S‐Box B) to inhibit HMGB1 by the mutation of the C residues to serine, which were injected into the NAc core after CPP training (Figure S3B,C). The behavioral results showed that cocaine‐induced CPP was suppressed by S‐Box B but not enhanced by C‐Box B (Figure 4a,b). We then infused C‐Box B into the NAc before CPP training. Interestingly, intra‐NAc infusion of peptide C‐Box B prior to cocaine administration disrupted the formation of cocaine memory at two different dosages of cocaine (5 and 15 mg/kg) (Figure 4c–f), which was consistent with the previous report that HMGB1 disrupted memory encoding in a novel object recognition test (Mazarati et al., 2011). These results reveal that cocaine directly binds to microglial TLR4 in the NAc and mediates the formation of cocaine‐associated memory.

### 3.5 The HMGB1–RAGE axis in the NAc is involved in the consolidation of cocaine‐related memory

**FIGURE 5:**
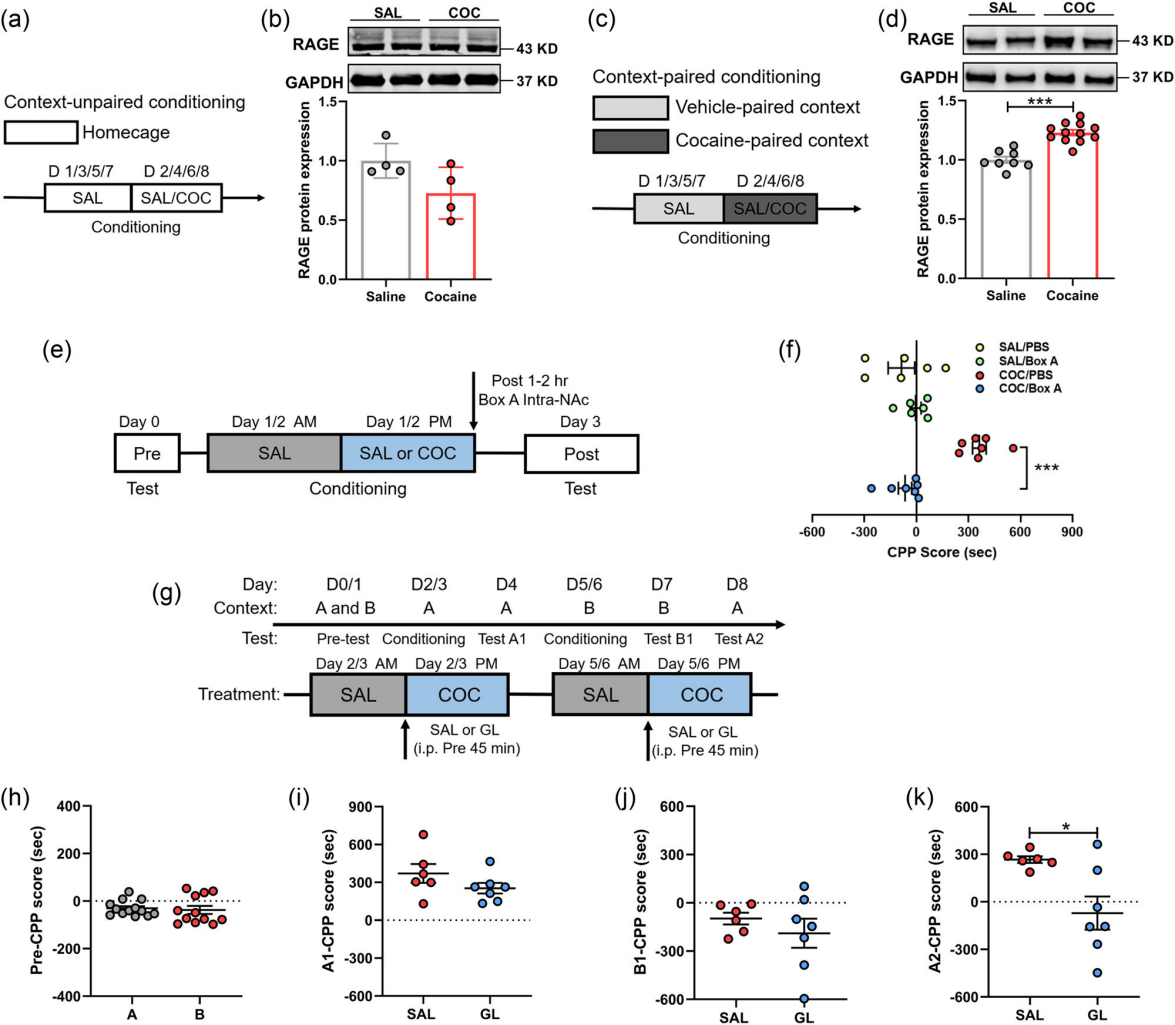
The high mobility group box‐1 (HMGB1)/receptor for advanced glycation end products (RAGE) axis in the nucleus accumbens (NAc) is required for consolidation of cocaine‐induced conditioned place preference (CPP). (a) Schematic representation of context unpaired conditioning. (b) Western blots showing the expression of RAGE in the NAc tissues after saline (SAL) or cocaine context unpaired conditioning (*n* = 4 rats per group). (c) Schematic representation of context paired conditioning. (d) Western blots showing the expression of RAGE in the NAc tissues after SAL or cocaine context paired conditioning (SAL: *n* = 8, cocaine [COC]: *n* = 12). (E and F) Timeline of the experiment (e) and the CPP score (f) after posttreatment with phosphate‐buffered saline (PBS), Box A into the NAc of rats (*n* = 6–7 rats per group). (g) Behavioral paradigm of a two‐phase CPP. (h) The baseline preferences in Contexts A and B (*n* = 12–13 rats per group). (i–k) The CPP score in test A1 (I) (*n* = 6–7 rats per group), test B1 (j) (*n* = 6–7 rats per group) and test A2 (k) (*n* = 6–7 rats per group). GL, glycyrrhizin. All data are presented as the mean ± SEM, with each point representing data from an individual. Two‐way ANOVA followed by Bonferroni's post hoc test for (f) and Student's t test for (b, d, h–k). *p < .05, ***p < .001. See Table S4 for detailed statistical information.

RAGE is another direct binding partner of HMGB1 (Andersson & Tracey, 2011; Harris et al., 2012), which is expressed mainly on microglia (Franklin et al., 2018). To assess whether HMGB1–RAGE axis was involved in the consolidation of cocaine‐induced CPP, we first examined the expression of RAGE in the NAc after CPP training. Following unpaired CPP, western blot analysis revealed that there were no significant differences in the expression of RAGE between the groups (Figure 5a,b). However, following paired CPP, the levels of RAGE were increased in the NAc of the paired context group compared with that of control group (Figure 5c,d).

Next, we designed the peptide of Box A, a specific antagonist to interrupt the interaction of HMGB1 and RAGE (LeBlanc et al., 2014; Muhammad et al., 2008). As the accumulation of extracellular HMGB1 was occurred after cocaine injection (Gao et al., 2020), Box A was microinjected into the NAc during 1–2 h after daily CPP training (Figure 5e). It was found that Box A impaired the cocaine‐induced CPP relative to PBS (Figure 5f). These results suggest that the injection of Box A into the NAc after CPP training session abolishes the expression of cocaine reward memory.

Considering that extracellular HMGB1‐dependent microglial activation in the NAc induced by cocaine exposure is occurred over 24 h after first cocaine administration, we asked whether HMGB1‐dependent microglial activation in the NAc contributed to the consolidation of cocaine reward memory. Previous studies have reported that two and four pairing sessions could induce CPP with the similar magnitude; however, cocaine‐induced CPP after four pairings would be greater than that after two pairings (Brabant et al., 2005; Tzschentke, 2007). Therefore, rats underwent a four‐pairing CPP here (first two pairing conditionings in Context A, last two pairing conditionings in Context B) (Figure 5g). There were no differences in the baseline preferences for both contexts (Figure 5h). However, after pretest conditioning, rats represented different preferences in the cue of Contexts A and B (Figure S4). Then, we used GL to interfere with HMGB1–RAGE signaling and suppress microglia activation. After first two pairing conditionings in Context A (in test A1), GL did not decrease the preference for cocaine‐paired box (Figure 5I). After last two pairing conditionings in Context B (in test B1), all rats showed no preference for cocaine‐paired box (Figure 5j). Then, in test A2, only SAL‐treated rats showed preference for cocaine‐paired box, and there was no preference for cocaine‐paired box in GL‐treated rats (Figure 5k). These findings indicate that HMGB1–RAGE axis‐dependent microglia activation is required for the consolidation of cocaine‐induced memory.

## 4 DISCUSSION

**FIGURE 6:**
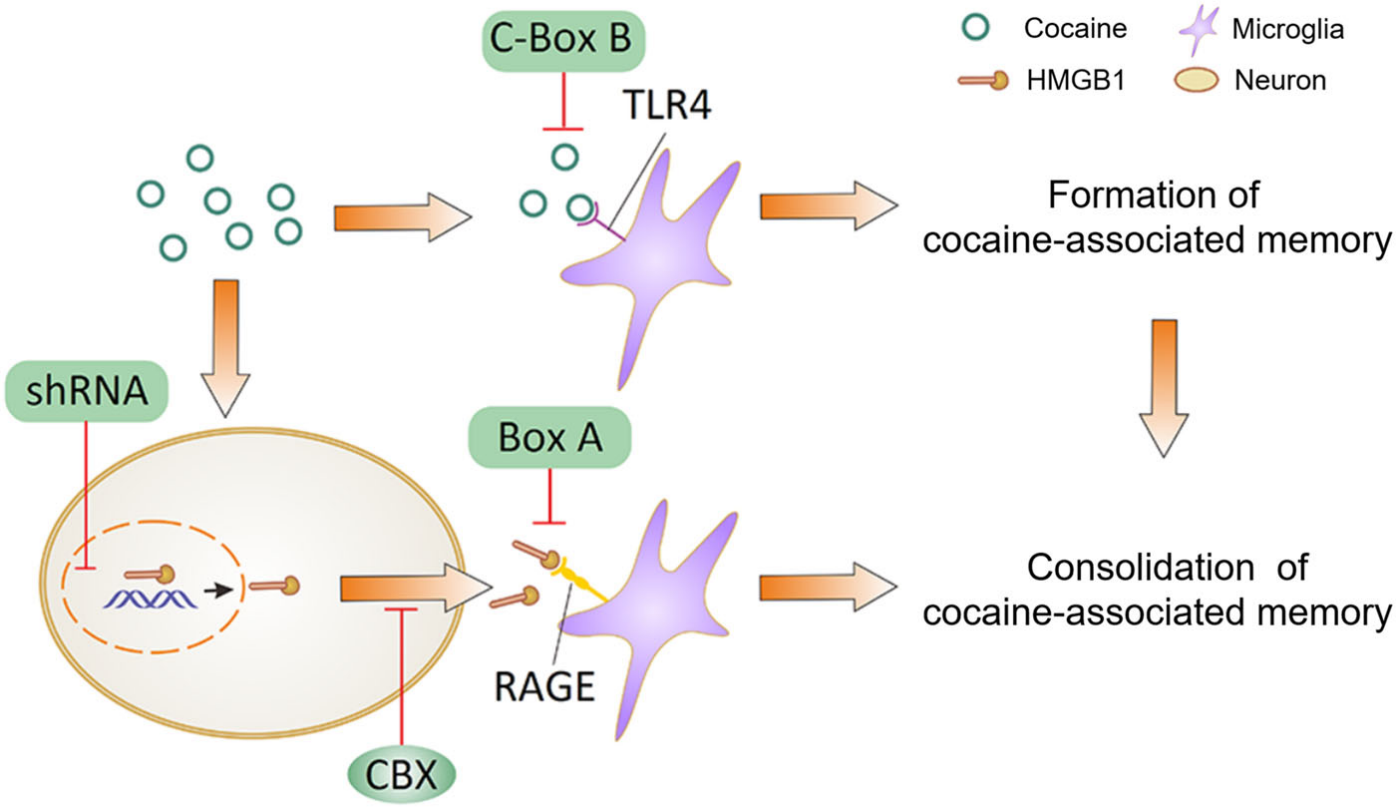
Diagram for the mechanism of early and late effects of cocaine‐induced memory. The formation of cocaine memory was mediated by cocaine binding directly to toll‐like receptor 4 (TLR4), and the consolidation of cocaine memory was induced by high mobility group box‐1 (HMGB1)‐receptor for advanced glycation end products (RAGE) axis. RAGE was engaged secondary to TLR4 after cocaine exposure and involved in the activation of microglia. There was a neural HMGB1‐dependent role for cocaine‐induced microglial activation in the nucleus accumbens (NAc) through RAGE, which contributed to the consolidation of cocaine reward memory.

In this study, we demonstrated for the first time that there were two distinct mechanisms underlying formation and consolidation of cocaine‐induced memory. Cocaine‐induced memory formation was mediated by binding cocaine directly to TLR4; however, cocaine‐induced memory consolidation was mediated by HMGB1–RAGE axis (Figure 6). Although TLR4 and RAGE shared common ligands and signal pathways, we found that RAGE was engaged secondary to TLR4 after cocaine exposure and involved in the activation of microglia. There was a neural HMGB1‐dependent role for cocaine‐induced microglial activation in the NAc through RAGE, but not TLR4, which contributed to the consolidation of cocaine reward memory. As synthetic HMGB1 fragments that specifically interfere with HMGB1–RAGE signaling after CPP training sessions could attenuate cocaine‐induced CPP, targeting HMGB1–RAGE signaling pathway might be an effective approach to ameliorate cocaine addiction.

As a DAMP, there are many pattern recognition receptors that can mediate effects of HMGB1 (e.g., TLRs, RAGE, and CD24) (Andersson & Tracey, 2011; Harris et al., 2012; Xue et al., 2021). As expected, both TLR4 and RAGE expressions were upregulated during cocaine action. Furthermore, both TLR4 and RAGE upregulations were reversed by HMGB1 silencing or HMGB1 blockade during cocaine‐induced CPP. Both HMGB1–RAGE and HMGB1–TLR4 signaling pathways were involved in the cocaine‐induced memory. However, the role of TLR4 and RAGE contributing to the onset of drug was different. First, the level of TLR4 was increased along with cocaine exposure, whereas only context‐paired CPP increased the level of RAGE in the NAc. Second, when we used exogenous synthetic peptide Box A to disturb the activity of endogenous HMGB1–RAGE signal (Maroso et al., 2010) after CPP training sessions, the cocaine‐induced CPP was ameliorated. However, the activation of HMGB1–TLR4 signaling pathway after CPP training sessions did not strengthen the cocaine reward memory. On the contrary, pretreatment with peptide C106‐Box B to mimic the binding of HMGB1 and TLR4 prevented cocaine‐induced CPP. The finding that C106‐Box B disturbed the binding of cocaine and TLR4 was consistent with the previous study that TLR4 agonist‐activated microglia in the NAc suppressed cocaine‐induced sensitization (Lewitus et al., 2016). Consequently, cocaine‐TLR4, but not HMGB1–TLR4, contributes to the formation of cocaine‐associated memory in the NAc (Figure 6).

The TLR4 signaling works rapidly within a few minutes, whereas HMGB1 acts as a delayed mediator of inflammation (Andersson & Tracey, 2011; Harris et al., 2012; Mao et al., 2022), which shows to be activated over 24 h after first cocaine injection (Gao et al., 2020). Then, we speculated that HMGB1–RAGE axis in the NAc might contribute to the late stage of cocaine reward memory. To verify this speculation, an experiment to test the incentive properties of conditioned stimulus (CS) was performed, and the primary CS could not be transferred to the secondary contextual cues in a CPP apparatus, but the HMGB1 inhibitor impaired the association of cocaine with primary CS during CPP training sessions in the secondary context. All above results support the fact that HMGB1–RAGE signaling is required for the consolidation of cocaine‐induced memory (Figure 6).

Cocaine can activate microglia directly and induce a plethora of cytokines and chemokines releasing, leading to the development of drug addiction (da Silva et al., 2023; Periyasamy et al., 2018). Our previous study demonstrates that intracellular HMGB1 regulates the formation of cocaine‐related memory by binding to glutamate receptor (Gao et al., 2020), however, whether the secretion of neural HMGB1 acts on microglia or contributes to cocaine addiction is largely unknown. A previous study has suggested that cocaine interacts with TLR4 on microglia directly (Northcutt et al., 2015). Parallel to these findings, our results also showed the increased amount of TLR4 protein in the NAc at 24 h after first cocaine injection. However, Iba1 and ED1, two markers of microglia in the NAc, did not show any changes at this time point, suggesting that there was a delay effect of microglia activation following cocaine exposure, which was consistent with previous study (Lewitus et al., 2016). On the other hand, suppression of microglia activation by Mino had no effects on the expression of HMGB1, but the HMGB1 inhibitor GL prevented the activation of microglia in the NAc during cocaine‐induced CPP. Thus, the delayed activation of microglia in the NAc during repeated cocaine exposure was dependent on HMGB1 signal. As the cocaine‐induced CPP was ameliorated after inhibition of microglial activation, and HMGB1–RAGE contributed to the late effect of cocaine, we proposed that there was a microglial RAGE‐dependent role for neural HMGB1 in cocaine‐induced CPP. Further work is needed to determine which cell types might be important for the behavioral effects of RAGE.

## 5 CONCLUSIONS

Our study demonstrates that the secretion of neural HMGB1 triggers the late effect of cocaine‐induced microglial activation by HMGB1–RAGE axis, which contributes to cocaine‐induced reward memory consolidation. As a late inflammatory mediator, HMGB1 can be released to extracellular region and activate microglia, mediating consolidation of cocaine reward memory. Therefore, therapeutic strategies targeting HMGB1 signaling, especially the HMGB1–RAGE axis, may be an effective approach to prevent the cocaine addiction.

## AUTHOR CONTRIBUTIONS

Jian Ye and Shuang‐Qi Gao performed most of the experiments and wrote the manuscript. Zi‐Cun Liu and Xi Chen assisted the addictive behavior. Jin‐Gang He assisted the behaviors of peptides. Zhuang‐Li Hu designed the project and revised the manuscript.

## CONFLICT OF INTEREST STATEMENT

The authors declare no conflicts of interest.

### PEER REVIEW

The peer review history for this article is available at https://publons.com/publon/10.1002/brb3.3457.

## Supporting information

Supporting Information

## Data Availability

The authors confirm that the data supporting the findings of this study are available within this article and from the corresponding author upon reasonable request.
