# Supplementary material for: The HMGB1–RAGE axis in nucleus accumbens facilitates cocaine‐induced conditioned place preference via modulating microglial activation
Source: Brain Behav. 2024 Mar 7;14(3):e3457. doi: 10.1002/brb3.3457 (PMC10918599; doi:10.1002/brb3.3457)
Supplement: Supplementary file 1 — Supporting Information [file BRB3-14-e3457-s001.docx]

**The HMGB1-RAGE axis in Nucleus Accumbens Facilitates Cocaine-Induced Conditioned Place Preference via Modulating Microglial Activation**

**Supplementary Methods and Materials**

**Drugs**

Cocaine hydrochloride (COC, purity > 98%) was obtained from Qinghai Pharmaceutical Factory (Qinghai Province, China). Minocycline hydrochloride (Mino), carbenoxolone (CBX) and glycyrrhizin (GL) were obtained from Sigma-Aldrich (St Louis, MO, USA). The information of Box A, C106-Box B and S106-Box B were listed on Supplementary Table 2.

**Systemic administration of drugs**

Rats received an intraperitoneal injection (i.p.) of either 30 mg/kg Mino (or 10, 20, 40 mg/kg CBX or 50 mg/kg GL) or equivolume of saline. Forty-five minutes later, rats received the second identical i.p. injection of either saline (SAL) or 15 mg/kg COC for CPP.

**Surgery and virus intracranial injections**

For stereotaxic injection of lentivirus (LV), rats were anesthetized with sodium pentobarbital (40 mg/kg) before surgery, and then placed in a stereotaxic apparatus, the lentiviruses (LV)-HMGB1-shRNA (5’-GAT CCG AAG CAC CCG GAT GCT TCTTTC AAG AGA AGA AGC ATC CGG GTG CTT CTT TTT TGG AAA-3’) (Genechem, Shanghai, China) were infused into the core of NAc (coordinates are in reference to bregma: AP + 1.7 mm, ML ± 2.5 mm, DV - 7.5 mm) through a 5 μl microsyringe, driven by a microinjection pump at a rate of 0.1 μl/min with volume of 2 μl (LV) or, rats were habituated to cages for at least 14 d (LV) before cocaine-induced CPP.

**Intra-NAc microinjection**

The intra-NAc injection was carried out as previously described (Gao et al., 2020). Briefly, two stainless steel cannulas with 15 mm length and 0.6 mm outside diameter were bilaterally implanted into the core of NAc region (AP + 1.7 mm, ML ± 2.5 mm, DV - 6.5 mm from the bregma and dural surface). To maintain patency, a stylus 0.5 mm longer than the guide cannula was inserted into the guide. After removal of the stylus from cannula, the drugs were injected into the NAc with a microsyringe (5 μl) connected by a PE-10 polyethylene tubing (10 cm) to a needle (0.5 mm in outside diameter, 0.5 mm longer than guide cannula), which was introduced into the brain region through the cannula fixing to the head of rat. The injection volume was set to 0.5 μl within a period of 1 min. The needle was withdrawn over a course of 10 min (Luo et al., 2015).

**Immunohistochemistry**

Tissue preparations were conducted as previously described (Gao et al., 2020). Briefly, the NAc slices were washed with PBS for 10 min × 3 times and blocked with PBS solution containing 3%-5% BSA, 0.3% Triton X-100, 2% normal donkey serum for 1 hr at RT. The slices were then incubated at 4 °C overnight with diluting primary antibody (Supplementary Table 3). After washing three times (5 min × 3 times) in PBS, slices were incubated at RT for 1 hr with secondary antibody (Supplementary Table 3). With another washing in PBS for 5 min × 3 times, slices were mounted with 50% glycerol/PBS and imaged using a laser confocal scanning microscope (FV500; Olympus, Tokyo, Japan).

Supplementary References

Gao SQ, Zhang H, He JG, et al. Neuronal HMGB1 in nucleus accumbens regulates cocaine reward memory. *Addict Biol.* 2020;25(2):e12739.

Luo Y, Zhou J, Li MX, et al. Reversal of aging-related emotional memory deficits by norepinephrine via regulating the stability of surface AMPA receptors. *Aging Cell.* 2015;14(2):170-179.

**Supplementary Figures**


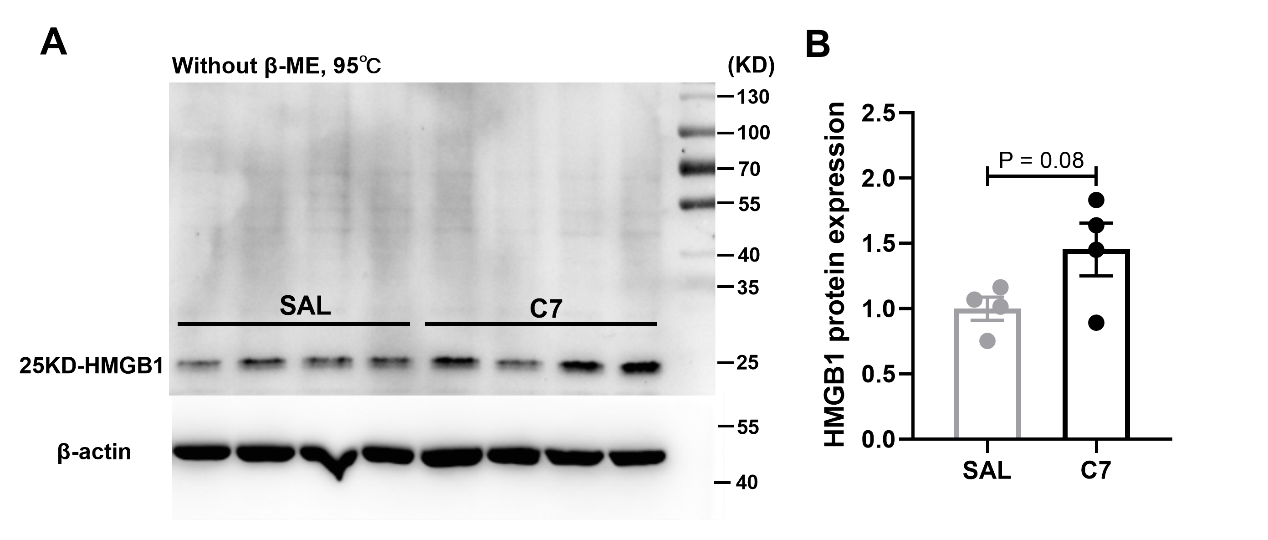


**Figure S1. Repeated cocaine exposure tends to increase the expression of oxidized-HMGB1 in the NAc.** (A-B) Western blotting results for processing samples in SDS-PAGE sample buffer without β-mercaptoethanol and heating at 95 °C for 5 min (n = 4 per group). SAL: saline; C7: seven daily injections of cocaine; 25 kDa-HMGB1: HMGB1 band size at 25 kDa; All-HMGB1: additional bands and 25 kDa-HMGB1. All data are presented as the mean ± SEM, with each point representing data from an individual. See Supplementary Table 4 for detailed statistical information.

**
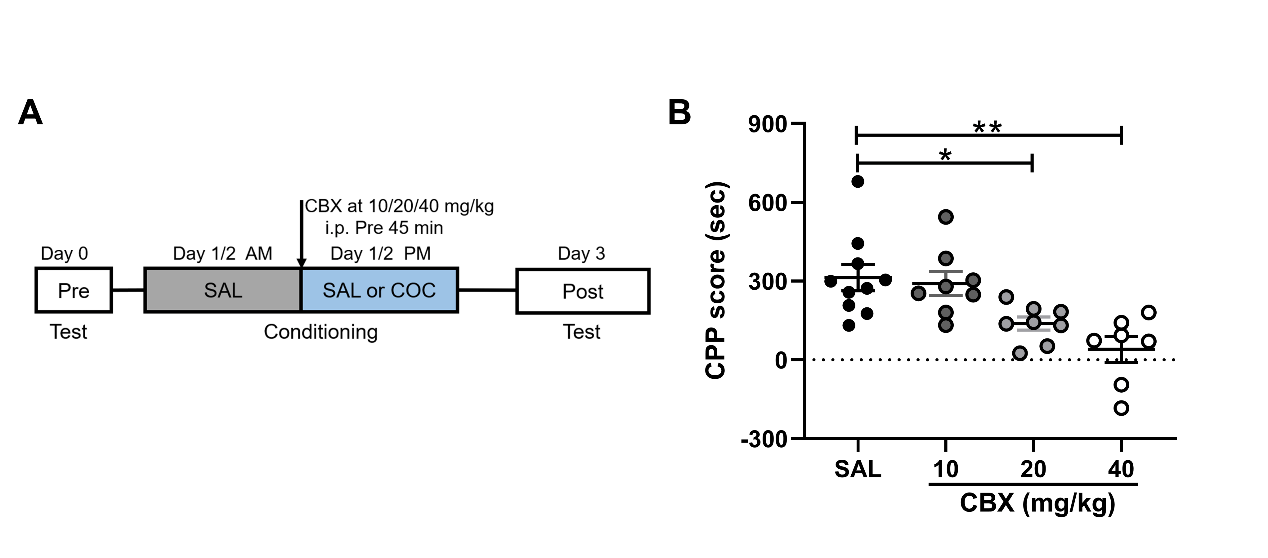
Figure S2. Pretreatment with carbenoxolone attenuates cocaine-induced CPP.** (A) Schematic representation of intraperitoneal injection of saline (SAL) or carbenoxolone (CBX) at 10, 20, 40 mg/kg into rats followed by training sessions. (B) The altered CPP score with CBX pretreatment (n = 7-10 per group). All data are presented as the mean ± SEM, with each point representing data from an individual. One-way ANOVA followed by Bonferroni’s post hoc test for (B). **p* < 0.05, ***p* < 0.01. See Supplementary Table 4 for detailed statistical information.


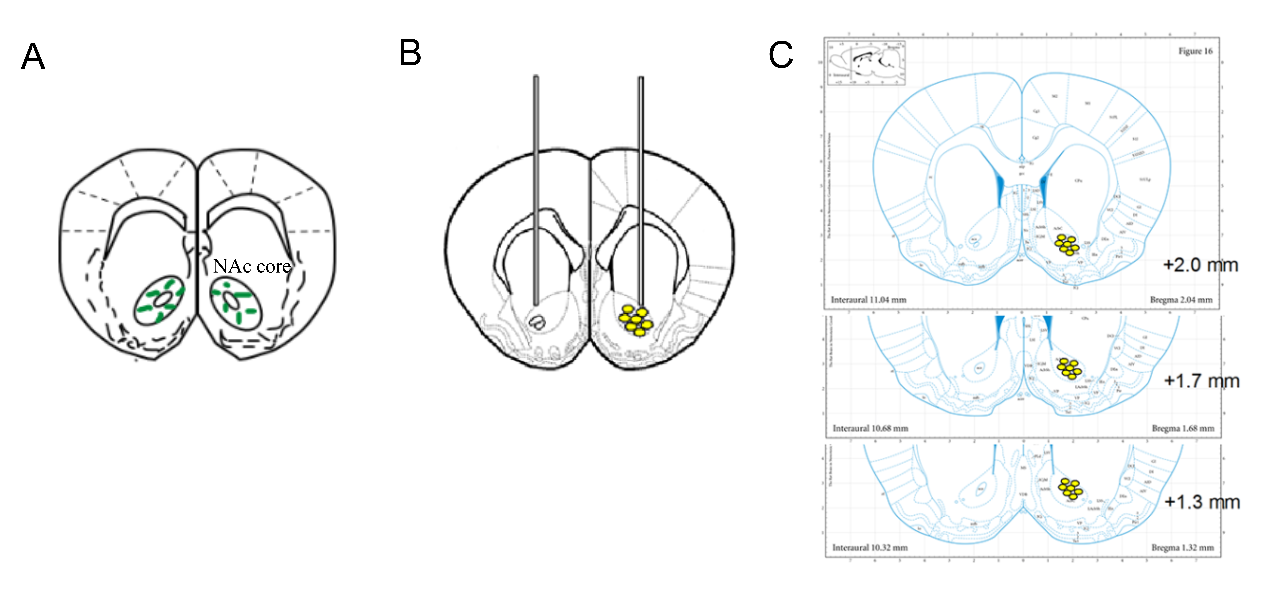


**Figure S3. The NAc tissue extraction and location of the injection cannulae tips within the NAc brain area.** (A) The schematic representation of the NAc core region assessed. (B-C) Representative schematic drawings of guide cannula tip positions (yellow dots) in the NAc core.


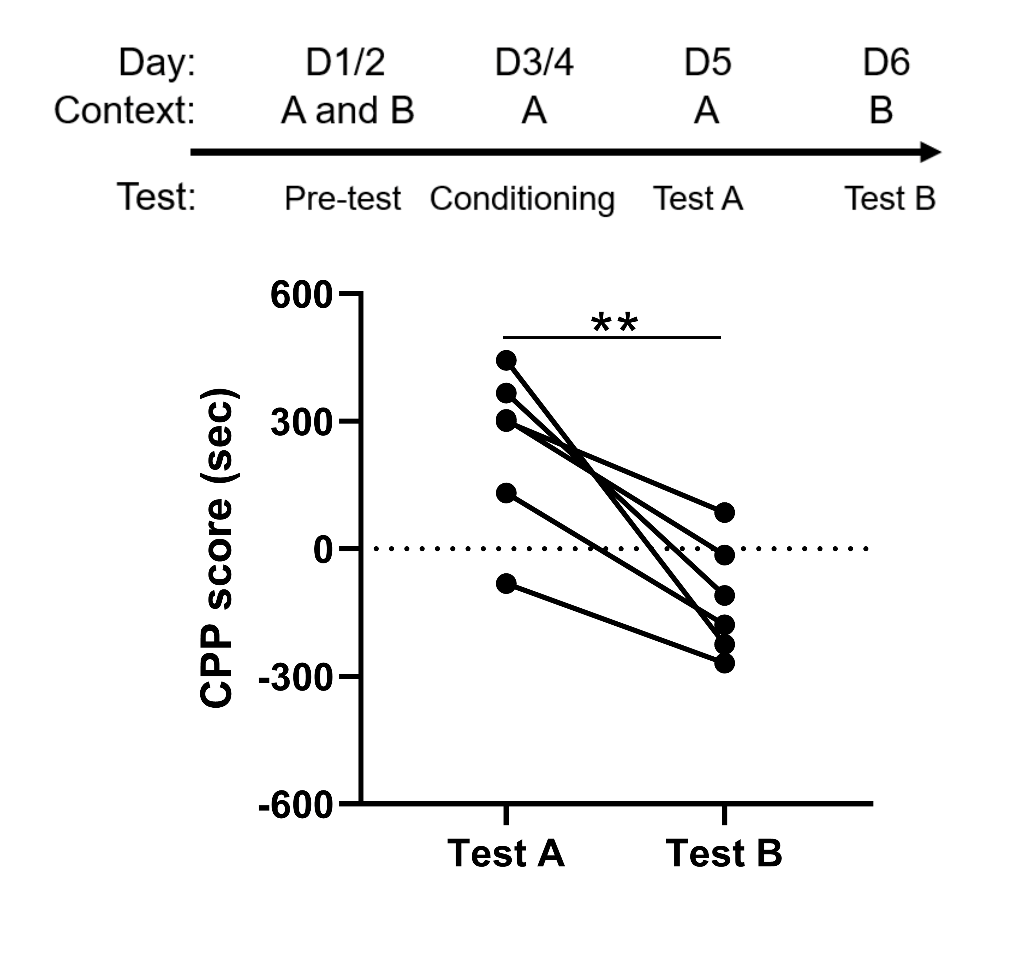


**Figure S4. The CPP score in Context A and Context B.** The behavioral results of CPP test showed the significant difference between Context A and Context B. All data are presented as the mean ± SEM, with each point representing data from an individual. Statistical analysis followed by student’s t test. ***p* < 0.01. See Supplementary Table 4 for detailed statistical information.


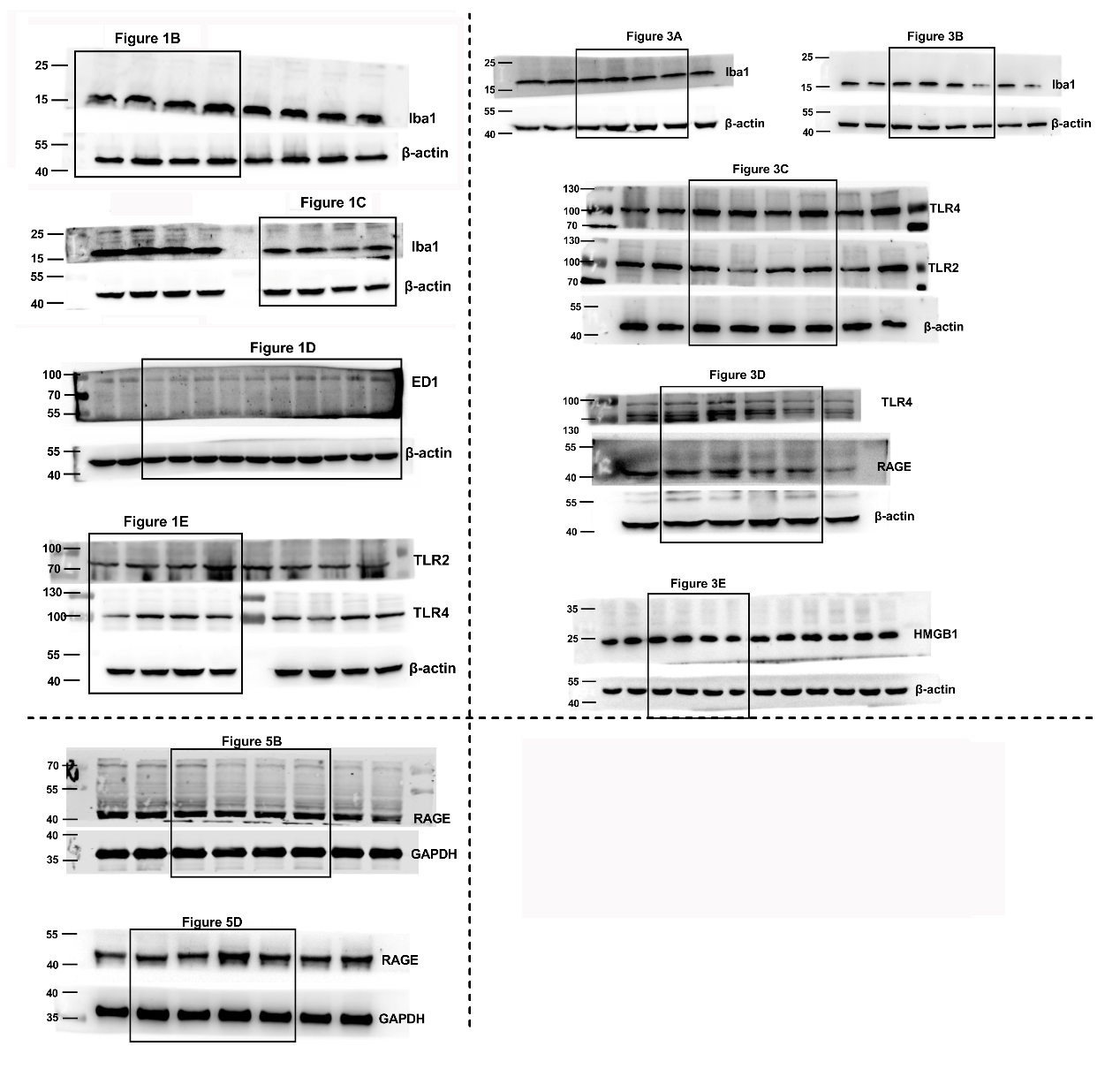


**Figure S5. Unedited blots for Figure 1B-E, 3A-E, 5B and 5D.**

**Supplementary Tables**

**Supplementary Table 1. The information of antibodies used in western blotting.**

| WB Antibody | Dilution | Host | Band size | Catalog # | Manufacturer |
| --- | --- | --- | --- | --- | --- |
| β-actin | 1:3000 | Mouse | 43 KD | sc-47778 | Santa Cruz, USA |
| GAPDH | 1:2000 | Mouse | 37 KD | sc-365062 | Santa Cruz, USA |
| HMGB1 | 1:1000 | Rabbit | 25 KD | ab18256 | Abcam, UK |
| Iba1 | 1:1000 | Goat | 17 KD | ab5076 | Abcam, UK |
| ED1 | 1:200 | Mouse | 90-100 KD | MAB1435 | Millipore, GER |
| TLR2 | 1:200 | Rabbit | 90-100 KD | sc-10739 | Santa Cruz, USA |
| TLR4 | 1:400 | Mouse | 96 KD | sc-293072 | Santa Cruz, USA |
| RAGE | 1:1000 | Rabbit | 43 KD | ab3611 | Abcam, UK |

**Supplementary Table 2. The sequences of different HMGB1 interfering peptides.**

| HMGB1 peptide | Sequence (position) | Manufacturer |
| --- | --- | --- |
| BoxA | MGKGDPKKPRGKMSSYAFFV  QTCREEHKKKHPDASVNFSEF  SKKCSERWKTMSAKEKGKFE  DMAKADKARYEREMKTYIPP  KGETKKKF (1-88) | HMGBiotech, Germany |
| S106-BoxB | FKDPNAPKRLPSAFFLFSSE (89-108) | GL Biochem (shanghai) Ltd, China |
| C106-BoxB | FKDPNAPKRLPSAFFLFCSE (89-108) | GL Biochem (shanghai) Ltd, China |

**Supplementary Table 3.** **The information of antibodies used in immunofluorescence.**

| IF Antibody | Dilution | Host | Catalog# | Manufacturer |
| --- | --- | --- | --- | --- |
| HMGB1 | 1:500 | Rabbit | ab18256 | Abcam, UK |
| Iba1 | 1:500 | Goat | ab5076 | Abcam, UK |
| Alexa 488 | 1:1000 | donkey anti-rabbit | A21206 | Thermo Fisher Scientific, USA |
| Alexa 594 | 1:1000 | donkey anti-goat | A11058 | Thermo Fisher Scientific, USA |

**Supplementary Table 4. Statistical analysis for Figure 1-5 and Figure S1-4**

| **Figure and numbers** | **Statistical analysis** | ***Post hoc tests*** | **Mean ± s.e.m.** |
| --- | --- | --- | --- |
| **1B** | **One-way ANOVA** | **Bonferroni’s Post hoc test** | **NAc：**  SAL = 1.000 ± 0.004  C1 = 1.050 ± 0.058  C3 = 1.172 ± 0.057  C7 = 1.167 ± 0.041 |
| **NAc：**  SAL =7  C1=7  C3=7  C7=7 | **NAc：**  F (3, 24) = 3.547,  p = 0.0295 | **NAc：**  SAL vs. C1, p > 0.9999  SAL vs. C3, p = 0.0410  SAL vs. C7, p = 0.0483 |  |
| **1C** | **One-way ANOVA** | **Bonferroni’s Post hoc Test** | **mPFC:**  SAL = 1.000 ± 0.000  C1 = 0.876 ± 0.084  C3 = 0.942 ± 0.120  C7 = 1.145 ± 0.175 |
| **mPFC:**  SAL =3  C1=3  C3=3  C7=3 | **mPFC:**  F (3, 8) = 1.009,  p = 0.4376 | **mPFC:**  SAL vs. C1, p > 0.9999  SAL vs. C3, p > 0.9999  SAL vs. C7, p > 0.9999 |  |
| **1D** | **One-way ANOVA** | **Bonferroni’s Post hoc Test** | **ED1:**  SAL = 1.000 ± 0.087  C3 = 0.909 ± 0.054  C7 = 1.577 ± 0.208 |
| **ED1**  SAL =4  C3=4  C7=4 | **ED1:**  F (2, 9) = 7.341,  p = 0.0129 | **ED1:**  SAL vs. C3, p > 0.9999  SAL vs. C7, p = 0.0275 |  |
| **1F** | **One-way ANOVA** | **Bonferroni’s Post hoc Test** | **TLR4:**  SAL = 1.000 ± 0.038  C1 = 1.189 ± 0.045  C3 = 1.224 ± 0.072  C7 = 1.186 ± 0.046 |
| **TLR4:**  SAL =5  C1=5  C3=4  C7=5 | **TLR4:**  F (3, 15) = 4.291,  p=0.0225 | **TLR4:**  SAL vs. C1, p = 0.0419  SAL vs. C3, p = 0.0213  SAL vs. C7, p = 0.0452 |  |
| **TLR2:**  SAL =3  C1=3  C3=4  C7=4 | **TLR2:**  F (3, 10) = 3.972  p=0.0421 | **TLR2:**  SAL vs. C1, p > 0.9999  SAL vs. C3, p > 0.9999  SAL vs. C7, p = 0.1281 | **TLR2:**  SAL = 1.000 ± 0.036  C1 = 0.972 ± 0.079  C3 = 0.942 ± 0.096  C7 = 1.235 ± 0.041 |
| **2B** | **Two-way ANOVA** | **Bonferroni’s Post hoc test** |  |
| SAL+SAL=8  Mino+SAL=6  SAL+COC=10  Mino+COC=10 | Mino**：**  F (1, 30) = 3.866, p=0.0586  COC：  F (1, 30) = 5.671, p=0.0238  Mino x COC**：**  F (1, 30) = 5.295, p=0.0285 | SAL+SAL vs. SAL+COC  p=0.0098  SAL+COC vs. Mino+COC  p=0.0133 | SAL+SAL=36.013±33.913  Mino+SAL=54.900±104.296  SAL+COC=300.130±28.385  Mino+COC=59.420±58.998 |
| **2D** | **Two-way ANOVA** | **Bonferroni’s Post hoc test** |  |
| SAL+SAL=7  CBX+SAL=6  SAL+COC=7  CBX+COC=7 | CBX**：**  F (1, 23) = 14.29, p=0.0010  COC：  F (1, 23) = 9.062, p=0.0062  CBX x COC**：**  F (1, 23) = 2.241, p=0.1480 | SAL+SAL vs. SAL+COC  p=0.0210  SAL+COC vs. CBX+COC  p=0.0054 | SAL+SAL=29.571±34.193  CBX+SAL=-75.333±81.823  SAL+COC=236.629±31.395  CBX+COC=-5.814±28.786 |
| **2F** | **Two-way ANOVA** | **Bonferroni’s Post hoc test** |  |
| SAL+SAL=7  GL+SAL=7  SAL+COC=7  GL+COC=7 | GL**：**  F (1, 24) = 13.26, p=0.0013  COC：  F (1, 24) = 1.685, p=0.2066  GL x COC**：**  F (1, 24) = 3.775, p=0.0639 | SAL+SAL vs. SAL+COC  p=0.1860  SAL+COC vs. GL+COC  p=0.0036 | SAL+SAL=24.543±38.683  GL+SAL=-94.800±97.997  SAL+COC=252.186±40.095  GL+COC=-140.086±83.827 |
| **3A** | **Unpaired t test** |  | **Iba1:**  SAL+COC = 1.000 ± 0.0624  GL+COC = 0.829 ± 0.0258 |
| SAL+COC = 4  GL+COC = 3 | **Iba1:**  t = 2.223,  p = 0.0769 |  |  |
| **3B** | **Unpaired t test** |  | **Iba1:**  SAL+COC = 1.000 ± 0.072  GL+COC = 0.647 ± 0.087 |
| SAL+COC = 7  GL+COC = 7 | **Iba1:**  t = 3.148,  p = 0.0084 |  |  |
| **3C** | **Unpaired t test** |  | **TLR4:**  SAL+COC = 1.000 ± 0.102  GL+COC = 0.633 ± 0.136 |
| **TLR4**:  SAL+COC = 11  GL+COC = 9 | **TLR4:**  t =2.202,  p =0.0410 |  |  |
| **TLR2**:  SAL+COC = 4  GL+COC = 4 | **TLR2:**  t =1.815,  p =0.1195 |  | **TLR2:**  SAL+COC = 1.000 ± 0.038  GL+COC = 1.139 ± 0.067 |
| **3D** | **Unpaired t test** |  | **TLR4:**  shGFP+COC =1.000 ±0.033  shHMGB1+COC = 0.717 ± 0.056 |
| **TLR4:**  shGFP+COC = 6  shHMGB1+COC = 7 | **TLR4:**  t =4.126,  p =0.0017 |  |  |
| **RAGE:**  shGFP+COC = 3  shHMGB1+COC = 3 | **RAGE:**  t =4.239,  p =0.0133 |  | **RAGE:**  shGFP+COC =1.000 ±0.020  shHMGB1+COC = 0.720 ± 0.063 |
| **3E** | **Unpaired t test** |  |  |
| **HMGB1:**  SAL+COC=10  Mino+COC=15 | **HMGB1:**  t =1.881,  p =0.0727 |  | **HMGB1:**  SAL+COC= 1.000±0.076  Mino+COC= 0.862±0.033 |
| **4B** | **Two-way ANOVA** | **Bonferroni’s Post hoc test** |  |
| SAL+PBS=5  SAL+C-Box B=6  COC+PBS=6  COC+S-Box B=7 | Box:  F (2, 30) = 7.963, p=0.0017  COC：  F (1, 30) = 23.73, p<0.0001  Box x COC**：**  F (2, 30) = 4.458, p=0.0202 | SAL+PBS vs. COC+PBS  p=0.017  COC+PBS vs. COC+S-Box B  p=0.0018  SAL+PBS vs COC+C-Box B  p=0.0220 | SAL+PBS=-22.300±62.149  SAL+C-BoxB=  -106.650±45.481  COC+PBS=366.250±65.983  COC+S-BoxB=  -80.386±97.756 |
| **4D** | **Unpaired t test** |  | PBS+COC = 317.80 ±99.45  C-BoxB+COC =-21.53 ±66.29 |
| PBS+COC = 5  C-Box B+COC = 4 | t = 2.674,  p = 0.0318 |  |  |
| **4F** | **Unpaired t test** |  |  |
| PBS+COC = 6  C-Box B+COC = 7 | t = 4.591,  p = 0.0008 |  | PBS+COC = 313.00 ±60.15  C-BoxB+COC=-151.0±77.85 |
| **5B** | **Unpaired t test** |  | **RAGE:**  SAL =1.000±0.0728  COC=0.727±0.109 |
| **RAGE:**  SAL = 4  COC= 4 | **RAGE:**  t = 2.081,  p = 0.0827 |  |  |
| **5D** | **Unpaired t test** |  | **RAGE:**  SAL =1.000±0.0273  COC=1.229±0.0235 |
| **RAGE:**  SAL = 8  COC= 12 | **RAGE:**  t = 6.292,  p <0.0001 |  |  |
| **5F** | **Two-way ANOVA** | **Bonferroni’s Post hoc test** |  |
| SAL+PBS=6  SAL+Box A=6  COC+PBS=7  COC+Box A=7 | Box A:  F (1, 22) = 12.88, p=0.0016  COC：  F (1, 22) = 16.20, p=0.0006  Box A x COC**：**  F (1, 22) = 27.82, p<0.0001 | SAL+PBS vs. COC+PBS  p<0.0001  COC+PBS vs. COC+Box A  p<0.0001 | SAL+PBS=-86.383±76.615  SAL+BoxA=-4.917±31.679  COC+PBS=362.957±38.959  COC+S-BoxB=  -65.300±38.392 |
| **5H** | **Unpaired t test** |  | A = -30.38 ±8.89  B = -37.38 ±16.93 |
| A = 13  B = 12 | t = 0.3739,  p =0.7119 |  |  |
| **5I** | **Unpaired t test** |  | SAL=370.70 ± 74.82  GL=253.60 ±41.83 |
| SAL=6  GL=7 | t =1.421,  p = 0.1831 |  |  |
| **5J** | **Unpaired t test** |  | SAL=-97.95 ± 36.38  GL=-189.30 ±90.37 |
| SAL=6  GL=7 | t =0.8805,  p = 0.3974 |  |  |
| **5K** | **Unpaired t test** |  | SAL=266.40 ±20.99  GL=-71.36 ±104.50 |
| SAL=6  GL=7 | t =2.931,  p =0.0137 |  |  |
| **S1B** | **Unpaired t test** |  | SAL=1.000 ±0.0878  C7=1.454±0.202 |
| SAL=4  C7=4 | t =2.058,  p =0.085 |  |  |
| **S2B** | **One-way ANOVA** | **Bonferroni’s Post hoc Test** | SAL = 313.70 ± 49.73  CBX (10mg/kg) = 290.90 ±45.14  CBX (20mg/kg) = 138.40 ± 25.16  CBX (40mg/kg) = 40.16 ± 49.46 |
| SAL=10  CBX(10mg/kg)=8  CBX(20mg/kg)=8  CBX(40mg/kg)=7 | F(3,29)=8.199  p=0.0004 | SAL vs. CBX (10mg/kg), p = 0.9679  SAL vs. CBX (20mg/kg), p = 0.0200  SAL vs. CBX (40mg/kg), p = 0.0005 |  |
| **S4** | **Unpaired t test** |  |  |
| Test A=6  Test B=6 | t =3.815,  p =0.0034 |  | Test A =243.90 ±77.50  Test B =-118.30±54.79 |
